# Validation of the Appendicitis Inflammatory Response (AIR) Score

**DOI:** 10.1007/s00268-021-06042-2

**Published:** 2021-04-06

**Authors:** Manne Andersson, Blanka Kolodziej, Roland E. Andersson

**Affiliations:** 1 Department of Biomedical and Clinical Sciences, Faculty of Health Sciences, Linköping University, Linköping, Sweden; 2 Department of Surgery, County Hospital Ryhov, 551 85 Jönköping, Sweden; 3 Department of Pathology, County Hospital Ryhov, County Council of Jönköping, Jönköping, Sweden

## Abstract

**Background:**

Patients with suspicion of appendicitis present with a wide range of severity. Score-based risk stratification can optimise the management of these patients. This prospective study validates the Appendicitis Inflammatory Response (AIR) score in patients with suspicion of appendicitis.

**Method:**

Consecutive patients over the age of five with suspicion of appendicitis presenting at 25 Swedish hospital’s emergency departments were prospectively included. The diagnostic properties of the AIR score are estimated.

**Results:**

Some 3878 patients were included, 821 with uncomplicated and 724 with complicated appendicitis, 1986 with non-specific abdominal pain and 347 with other diagnoses. The score performed better in detecting complicated appendicitis (ROC area 0.89 (95% confidence interval (CI) 0.88–0.90) versus 0.83 (CI 0.82–0.84) for any appendicitis, *p* < 0.001), in patients below age 15 years and in patients with >47 h duration of symptoms (ROC area 0.93, CI 0.90–0.95 for complicated and 0.87, CI 0.84–0.90 for any appendicitis in both categories). Complicated appendicitis is unlikely at AIR score <4 points (Negative Predictive Value 99%, CI 98–100%). Appendicitis is likely at AIR score >8 points, especially in young patients (positive predictive value (PPV) 96%, CI 90–100%) and men (PPV 89%, CI 84–93%).

**Conclusions:**

The AIR score has high sensitivity for complicated appendicitis and identifies subgroups with low probability of complicated appendicitis or high probability of appendicitis. The discriminating capacity is high in children and patients with long duration of symptoms. It performs equally well in both sexes. This verifies the AIR score as a valid decision support.

*Trial registration number*

https://clinicaltrials.gov/ct2/show/NCT00971438

## Introduction

In unselected patients with suspicion of acute appendicitis, the prevalence is typically about 30% and the clinical presentation varies from a mild to an overt septic condition. The management of these patients is resource consuming. Non-productive admissions and surgical explorations are common, indicating a need for improvement [1, 2].

The clinical diagnosis is the basis for the management but is commonly a non-systematic and subjective assessment of history, symptoms and signs, eventually supplemented by laboratory tests. Diagnostic imaging is increasingly used but is not universally available in all settings and its optimal role is controversial. As routine imaging may give high rates of false positive and false negatives in groups of patients with low and high prevalence of appendicitis, respectively, the use of imaging should be tailored to the patients pre-test probability of appendicitis [3–5]. Selective use of CT is also motivated to reduce ionising radiation exposure and potential risk of cancer induction [6]. Low dose CT or staged imaging, starting with ultrasound and using CT only in patients with unclear ultrasound result, may decrease this risk [7, 8].

**Fig. 1:**
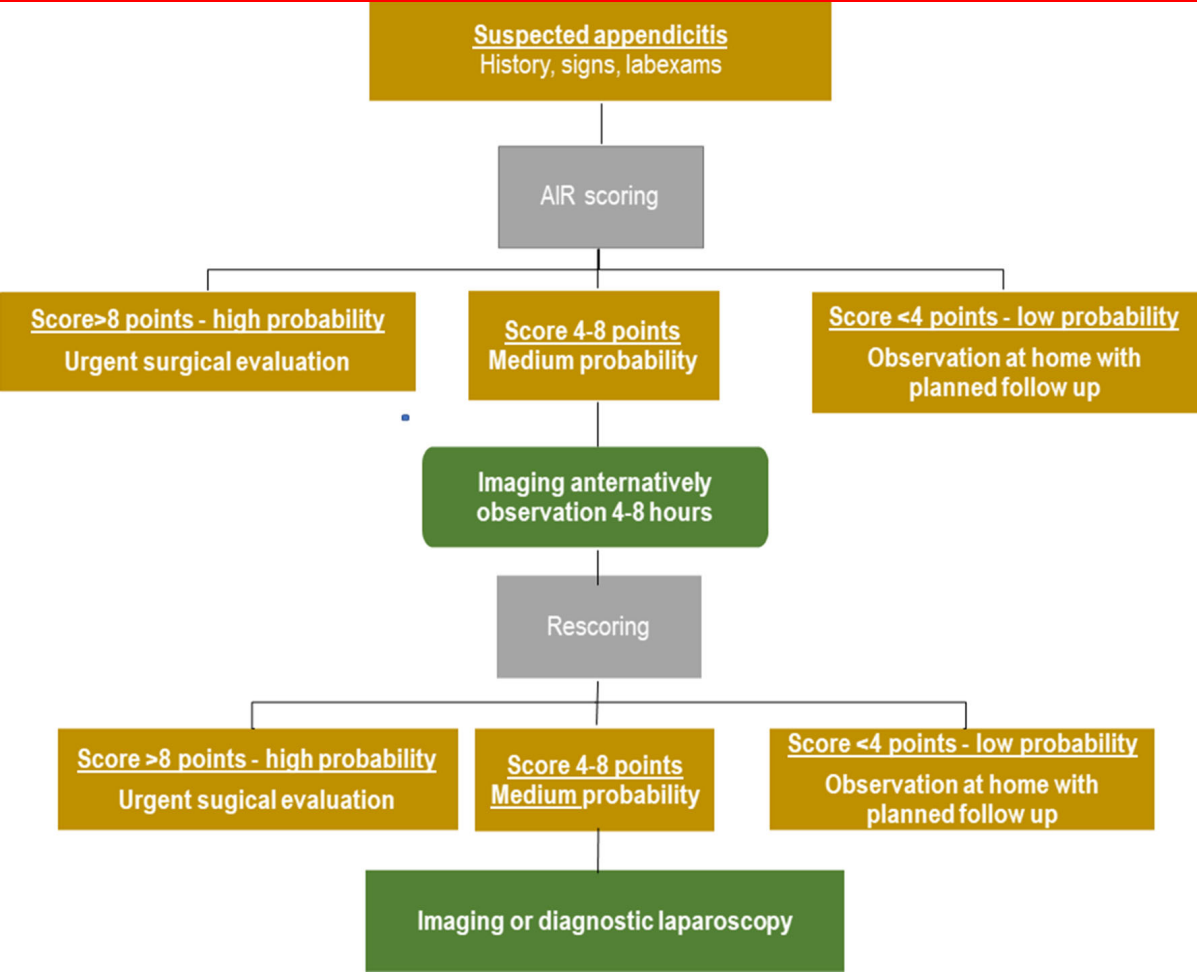
Proposed algorithm with risk-stratification based on the AIR-score. Compared to the original the low cutoff point is changed to <4 as a result of the present study

Risk-stratification based on a clinical score can be used to optimise the selection of patients for urgent surgical evaluation, diagnostic imaging, in-patient or out-patient observation. In a previous study, the prospective implementation of an algorithm based on the Appendicitis Inflammatory Response (AIR) score (Fig. 1) led to a reduction in unnecessary hospital admissions and a decreased use of diagnostic imaging [9, 10]. The present study is an in-depth validation of the AIR-score. We hypothesise that the AIR-score is a suitable decision support for the management of patients with suspected appendicitis.

## Patients and Methods

### Study design and setting

The present study is based on data from the STRAPPSCORE study (STRuctured management of patients with suspicion of APPendicitis using a clinical SCORE) which is a prospective interventional multicentre study with 25 participating Swedish hospitals (eight university hospitals, eight county hospitals, and nine general hospitals) including patients between September 2009 and January 2012. The main report from the STRAPPSCORE study has been published elsewhere [10].

### Data collection

Consecutive patients aged over five years presenting with low abdominal pain of less than 5 days duration suggestive of appendicitis were considered for inclusion. The AIR score parameters (right lower quadrant pain, intensity of rebound tenderness or muscular defence, CRP concentration, WBC count, proportion of neutrophils, body temperature, and history of vomiting) were prospectively registered. Duration of symptoms and the level of experience of the physician managing the patient on arrival were noted. The use of diagnostic imaging (ultrasound, US, and/or computerised tomography, CT), any surgical intervention, per-operative and discharge diagnoses, and use of antibiotics, were noted at the discharge.

The study was conducted in two phases. During the baseline phase, the AIR score parameters were recorded prospectively but the score was not determined, and the patients were managed according to the local standards. During the intervention phase, the AIR score sum is calculated (Table 1), and the physician was instructed to follow the proposed algorithm (Fig. 1). The present study includes the AIR score sum from both phases.

**Table 1:** Appendicitis Inflammatory Response (AIR) score, 0–12 points

Item	Scoring point
Vomiting	1
Pain in right inferior fossa	1
*Rebound tenderness or muscular defence*	
Light	1
Medium	2
Strong	3
Body temperature ≥38.5 °C	1
*White blood cell count*	
10.0–14.9 * 109/L	1
≥15.0 * 109/L	2
*Proportion polymorphonuclear leucocytes*	
70–84%	1
≥85%	2
*C-reactive protein concentration*	
10–49 mg/L	1
≥50 mg/L	2

Seven variables are assessed and scored accordingly. After the revision proposed in this report a score 0–3 points suggest low probability, a score 4–8 medium probability and a score 9–12 high probability

The AIR score sum defines three groups: low probability (<5 points), medium probability (5–8 points), and high probability (>8 points) [9]. At high probability the algorithm recommends immediate evaluation for eventual abdominal exploration. At low probability outpatient management with a planned follow-up within 24 h is proposed. In the STRAPSCORE study, the medium probability group was randomised to immediate imaging or a period of in-hospital observation followed by rescoring and selective imaging.

### Diagnosis

Participating surgeons were instructed to send all removed appendices for histopathological examination. All participating pathologists were blinded to the AIR-score sum and instructed to report about the presence or absence of transmural infiltration of neutrophils and transmural tissue necrosis. If a collection of pus surrounding the appendix or a perforation with free peritonitis was identified during surgery, the appendix is considered perforated. Patients that were diagnosed with an appendiceal abscess or phlegmone by imaging and treated with antibiotics and eventual drainage were classified as complicated appendicitis. The criteria for uncomplicated appendicitis is transmural neutrophil invasion. Complicated appendicitis is defined as presence of transmural necrosis or perforation. To obtain a standardised histopathological diagnosis, the excised appendices from patients in the high and low probability groups were re-examined by one consultant pathologist. Patients with other, non-appendicitis diagnoses, are included among the non-appendicitis group in all the following estimations.

All computerised tomography (CT) scans have been re-examined by radiologists blinded to the original report. A diagnosis of appendicitis is accepted also for non-operated patients when an appendicitis diagnosis was consistently reported in both the original report and the repeat examination of the CT study.

### Follow-up

All patients were followed up for a minimum of 30 days through linkage with the Swedish national patient register using the Swedish national identification number, unique to all Swedish citizens [11]. Discharged patients with an operation for appendicitis at any Swedish hospital within seven days after the index admission are considered a *missed appendicitis*, and the outcome of the patient was changed according to the appendectomy diagnosis.

### Statistical analysis

#### Diagnostic properties of the AIR score

We use the receiver operating characteristic (ROC) area to analyse the discriminating capacity for any appendicitis (i.e. uncomplicated or complicated appendicitis) vs no appendicitis, and for complicated appendicitis vs no appendicitis. We estimate sensitivity, specificity, and predictive values at each score point and at the low and high cut offs, respectively. We report results for pre-defined subgroups of patients according to age, sex, duration of symptoms, and competence of the physician.

#### Missing values

The dataset contained missing values. Little’s test for the assumption of covariate-dependent missingness was not significant suggesting that multiple imputation is applicable to avoid biased results [12]. We used multiple chained equation imputation to replace missing values [13]. Patients with missing values in more than two scoring variables were excluded.

Categorical variables were compared by means of the *χ*^2^ test, and continuous variables using t-test or Mann–Whitney U test as appropriate. Significance is defined as a two-tailed *p*-value <0.05 for all comparisons. The data were analysed using Stata 15, StataCorp. 2015. *Stata Statistical Software: Release 15*. College Station, TX: StataCorp LP.

The study was approved by the Linköping University regional ethics committee (M15-09 and 2011/375-32) and was registered at ClinicalTrials.gov (NCT00971438).

## Results

### Study population

**Fig. 2:**
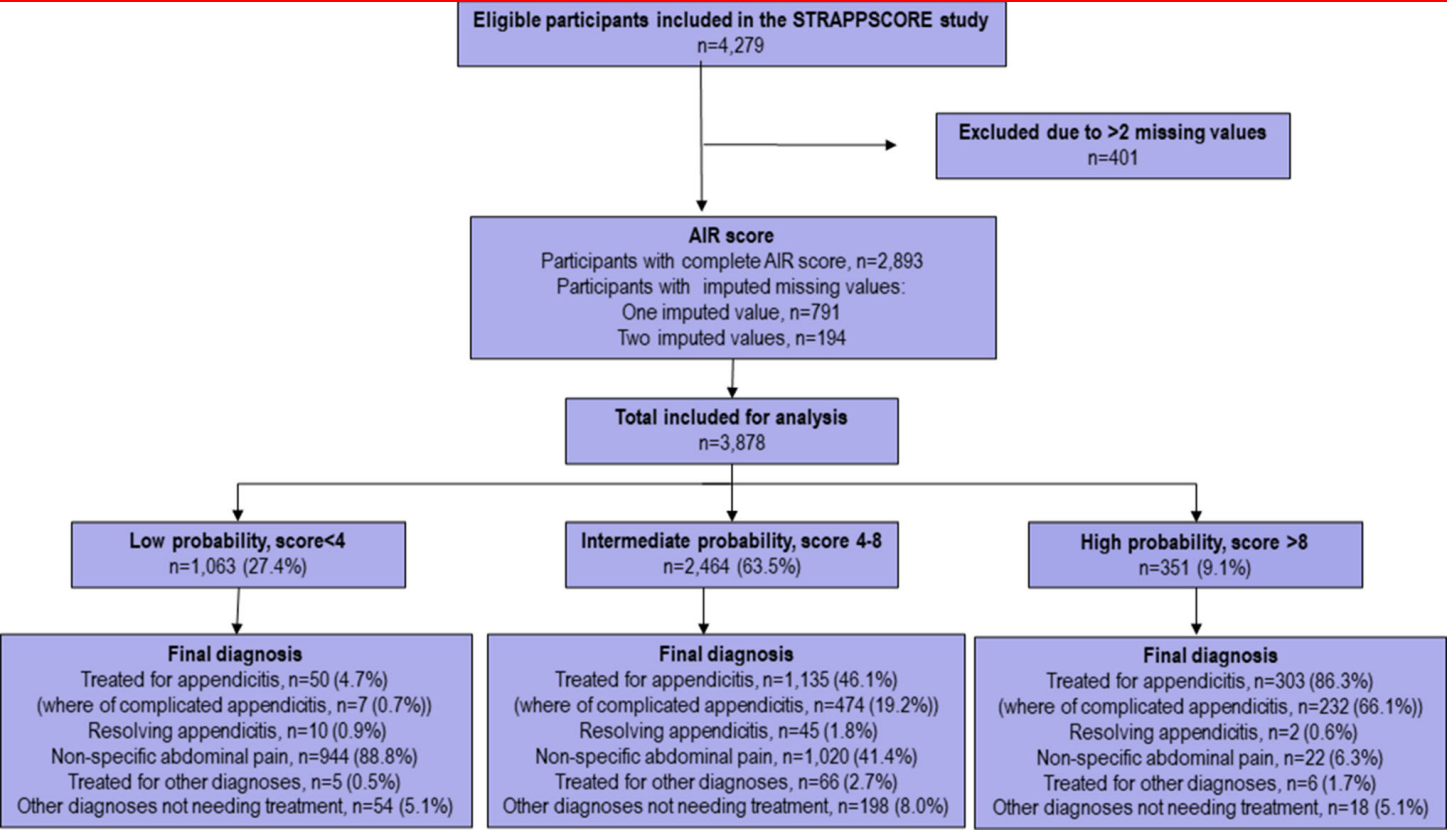
STARD-diagram of the flow of participants in the study

A total of 4279 patients were included in the STRAPPSCORE study (Fig. 2). Some 401 patients with more than two missing AIR score parameters were excluded, leaving 3878 patients for analysis. (Table 2) Patients with non-specific abdominal pain (NSAP) was the largest group (1986, 51.2%). Some 821 (21.2%) had uncomplicated and 724 (18.7%) complicated appendicitis and 347 (8.9%) had other diagnoses. Appendicitis was most common among men and NSAP, and other diagnoses were more common among women.

**Table 2:** Demography and characteristics of the included patients with suspicion of appendicitis

Characteristic	Total	Abdominal pain	Non-specific	Appendicitis	Other
			Uncomplicated	Complicated	Diagnoses
Numbers (%)	3878	1986 (51.2)	821 (21.2)	724 (18.7)	347 (8.9)
Age, median (IQR)	26.1 (18.2–40.3)	23.4 (17.6–34.9)	26.5 (18.5–38.7)	34.2 (19.9–50.2)	34.7 (21.5–54.1)
Males (%)	1802 (46.5)	751 (37.8)	483 (58.8)	423 (58.4)	145 (41.8)
Females (%)	2076 (53.5)	1235 (62.2)	338 (41.2)	301 (41.6)	202 (58.2)
Duration of symptoms, hours (IQR)	24 (12–48)	24 (10–48)	20 (12–30)	24 (14–48)	24 (12–48)
One missing value	791 (20.4%)	401 (20.2)	176 (21.4)	138 (19.1)	76 (21.9)
Two missing values	194 (5.0%)	120 (6.0)	38 (4.6)	29 (4.0)	7 (2.0)
Imaging	1788 (37.8)	606 (30.5)	496 (60.4)	412 (56.9)	274 (78.9)

One or two missing AIR score parameters were subsequently imputed in 985 (25.4%) patients. Missing values were most common for the proportion of neutrophils (*n* = 569 or 15% of total) followed by body temperature (*n* = 321 or 8% of total).

### The diagnostic performance of the AIR score

#### ROC area

The AIR score has a higher discriminating capacity for complicated appendicitis (ROC area 0.89 vs. 0.83 for any appendicitis, *p* < 0.001) (Table 3). For the diagnosis of any appendicitis, it performs best in patients below age 15 years (ROC area 0.87) and in patients with over 47 h duration of symptoms (ROC area 0.86). The corresponding results for complicated appendicitis are 0.92 and 0.93. It performs equally well in both sexes and irrespective of the examiner’s competence.

**Table 3:** Discriminating capacity of the AIR-score overall, in subsets of patients and according to the examiner’s competence, expressed as the ROC area

Characteristics	ROC area*			ROC area*		
	Appendicitis	95% CI	*p*-value	Complicated	95% CI	*p*-value
All patients	0.83	0.82–0.84		0.89	0.88–0.90	<0.001**
*Patients sex*			0.18			0.16
Women	0.84	0.82–0.85		0.90	0.88–0.91	
Men	0.82	0.80–0.84		0.88	0.86–0.90	
*Patients age*			0.001			< 0.001
<15 years	0.87	0.84–0.90		0.93	0.90–0.95	
15–39 years	0.83	0.81–0.84		0.89	0.88–0.91	
≥40 years	0.79	0.76–0.82		0.84	0.82–0.87	
*Duration of symptoms*			0.001			<0.001
<12 h	0.80	0.77–0.83		0.84	0.80–0.88	
12–23 h	0.81	0.78–0.84		0.86	0.83–0.89	
24–47 h	0.83	0.80–0.85		0.89	0.86–0.91	
>47 h	0.87	0.85–0.89		0.93	0.91–0.95	
*Examiners competence*			0.61			0.15
Interns	0.82	0.80–0.84		0.88	0.86–0.90	
Residents	0.83	0.81–0.85		0.90	0.88–0.92	
Specialists	0.82	0.79–0.85		0.86	0.83–0.90	

^*^ROC is Receiver Operating Curve

^**^p-value for the comparison of the ROC are for all appendicitis and complicated appendicitis

#### Validation of the cut-off points

In the original design study the AIR score obtained a sensitivity of 100% at the low cut-off point ≥5. In the present study, the sensitivity for complicated appendicitis is 96.1% at this cut-off point (Table 4). An adjustment of the low cut-off point to ≥4 points, which gives a sensitivity for complicated appendicitis of 99.0%, is therefore motivated. For the high probability group, the present study obtains an almost identical specificity at the high cut-off point ≥9 (98.0% vs. 99.0%).

**Table 4:** Distribution of patients over the AIR-score according to the final diagnosis, and corresponding diagnostic characteristics at all cut-off points

Score points	Numbers according to diagnosis								
	NSAP	Appendicitis		Other diagnoses	Total	Cut off points	Sensitivity		Specificity
		Uncomplicated	Complicated				Appendicitis (%)	Complicated (%)	Appendicitis (%)
0	60	0	0	2	62	≥0	100.0	100.0	0.0
1	195	2	1	6	204	≥1	100.0	100.0	2.7
2	323	12	0	21	356	≥2	99.8	99.9	11.3
3	366	39	6	30	441	≥3	99.0	99.9	26.0
4	364	83	25	45	517	≥4	96.1	99.0	43.0
5	292	156	75	62	585	≥5	89.1	95.6	60.5
6	210	183	114	64	571	≥6	74.2	85.2	75.7
7	104	160	137	52	453	≥7	55.0	69.5	87.4
8	50	116	131	41	338	≥8	35.7	50.6	94.1
9	15	55	116	15	201	≥9	19.7	32.5	98.0
10	6	12	76	5	99	≥10	8.7	16.4	99.3
11	1	3	39	1	44	≥11	3.0	5.9	99.8
12	0	0	4	3	7	≥12	0.3	0.6	99.9
Total	1 986	821	724	347	3 878				

A new low cut-off point >  = 4 is proposed as the original cut-off point >  = 5 has insufficient sensitivity for complicated appendicitis

#### Sensitivity, specificity, and predictive values

##### Low probability group

The low probability group aims to rule out patients with advanced appendicitis to safely practice outpatient observation and planned repeat examination. Some 1063 patients (27.4%) were classified as low probability with AIR-score <4 points. Seven patients (0.7%) had a final diagnosis of complicated appendicitis and another 4.7% had appendectomy for appendicitis in this group (Table 5). This corresponds to a sensitivity of 99% and a Negative Predictive Value (NPV) of 99% for complicated appendicitis, and a sensitivity of 96% and NPV of 94% for any appendicitis (Table 6). The NPV for complicated appendicitis was higher in women (100% vs. 98% for men, *p* = 0.042) but did not differ depending on age, examiners competence or duration of symptoms (Table 6). One patient in this group needed surgical treatment for other diagnosis (volvulus), and 23 patients (2.2%) had a non-therapeutic exploration. Ten patients with CT verified appendicitis resolved without treatment.

**Table 5:** Distribution of outcome in the three risk groups according to the AIR score

Outcome	AIR score					
	0–4 points No. %		5–8 points No. %		9–12 points No. %	
*Non-specific abdominal pain*						
No treatment	926	87.1	870	35.3	8	2.3
Antibiotics	2	0.2	29	1.2	4	1.1
Negative appendectomy	16	1.5	121	4.9	10	2.8
*Appendicitis*						
No treatment	10	0.9	45	1.8	2	0.6
Antibiotics	0	0	30	1.2	7	2.0
Appendectomy	50	4.7	1 105	44.8	296	84.3
(where of complicated appendicitis)	7	0.7	474	19.2	232	66.1
*Other diagnoses*						
No treatment	47	4.4	182	7.4	15	4.3
Antibiotics	4	0.4	44	1.8	4	1.1
Treated with surgery	1	0.1	22	0.9	2	0.6
Non-therapeutic abdominal exploration	7	0.7	16	0.6	3	0.9
Total non-therapeutic abdominal exploration	23	2.2	137	5.6	13	3.7
Imaging	230	21.6	1370	55.6	166	47.3
Total	1063	100	2464	100	351	100

The total number of non-therapeutic abdominal explorations includes all negative appendectomies and all abdominal explorations for other diagnosis not leading to any change in treatment

**Table 6:** Diagnostic properties at the low probability zone

Characteristics	AIR score 0–4					
	Complicated appendicitis					
	NPV	95% CI	*p*-value	Sensitivity	95% CI	*p*-value
All patients	99	98–100		99	98–100	
*Patients sex*			0.042			0.48
Women	100	99–100		99	98–100	
Men	98	97–100		98	97–100	
*Patients age*			0.44			0.85
<15 years	99	97–100		98	94–100	
15–39 years	99	98–100		98	97–100	
≥40 years	99	97–100		99	98–100	
*Duration of symptoms*			0.46			0.10
<12 h	99	98–100		97	94–100	
12–23 h	98	96–100		98	96–100	
24–47 h	99	98–100		100	99–100	
>47 h	99	98–100		99	97–100	
*Examiners competence*			0.52			0.87
Interns	99	98–100		99	97–100	
Residents	99	98–100		99	98–100	
Specialists	98	96–100		98	95–100	

NPV and sensitivity for complicated appendicitis is presented as not missing complicated appendicitis is most important to safely practice observation

### High probability group

The aim for the high probability group is to select patients for urgent surgical evaluation for eventual operation avoiding negative appendectomy. Some 351 patients (9.1%) are classified to the high probability group with AIR-score >8, of which 232 (66%) had complicated appendicitis (32.5% of all complicated appendicitis) (Table 5). The specificity for all appendicitis is 98% overall and slightly higher in children under age 15 years (99%) and patients with short duration of symptoms (99%). The positive predictive value (PPV) for any appendicitis is 86%. (Table 7) Twenty-four patients (6.8%) had another diagnosis. Two of them had surgical treatment—one for Crohns disease and one for rectal cancer. Thirteen patients (3.7%) had a non-productive abdominal exploration.

**Table 7:** Diagnostic properties in the high probability zone

Characteristics	AIR score 9–12					
	Any appendicitis					
	PPV	95% CI	*p*-value	Specificity	95% CI	*p*-value
All patients	86	83–90		98	97–99	
*Patients sex*			0.25			0.31
Women	84	78–90		98	97–99	
Men	89	84–93		98	97–99	
*Patients age*			0.18			<001
<15 years	96	90–100		99	98–100	
15–39 years	86	81–92		99	98–99	
≥40 years	83	76–89		95	94–97	
*Duration of symptoms*			0.74			0.047
<12 h	84		71–98	99	98–100	
12–23 h	89		81–97	98	97–100	
24–47 h	86		79–93	97	95–98	
>47 h	87		80–93	98	96–99	
*Examiners competence*			0.49			0.23
Interns	87	81–93		98	97–99	
Residents	88	82–94		98	97–99	
Specialists	83	74–92		97	95–99	

PPV and Specificity for any appendicitis is given as the focus is avoiding negative appendectomy

## Discussion

This large multicenter study verifies the AIR-score as a valid and reproducible instrument with high discriminating capacity especially for advanced appendicitis. It defines groups of patients with low, medium, and high probability of appendicitis with high sensitivity and specificity. A large proportion (47.5%) of the patients without appendicitis were assigned to the low risk group and 32.5% of all complicated appendicitis were assigned to the high risk group, showing its utility as basis for a safe risk-adapted management that can help in identifying patients in need of urgent surgical evaluation and minimising unproductive hospital admissions and abdominal explorations.

The clinical diagnosis and diagnostic imaging are the pillars in modern management of patients with suspicion of appendicitis, but the optimal management algorithm is still controversial. Routine imaging in unselected patients is not recommended because of the high frequency of false-positive and false-negative diagnosis in patients with low or high prevalence of appendicitis, respectively [3–5, 14]. Routine CT scanning in unselected patients with a mean prevalence of 32% will give an estimated PPV of only 70% [7]. Imaging will thus over diagnose appendicitis in patients with a low clinical probability and cannot rule out appendicitis in patients with high clinical probability. A meta-analysis of the cost-effectiveness of imaging strategies in children concluded that imaging is not cost-effective for patients with a risk of appendicitis <16% or >95% and that the imaging approach should be tailored on the basis of a patient’s pretest probability of appendicitis [15].

Many algorithms propose a risk-differentiated strategy but do not specify how the risk can be determined or only give a general reference to “typical history and clinical findings”. Clinical scoring systems are instruments to determine the probability of appendicitis in the individual patient [16]. The AIR score is based on mainly objective inflammatory markers which may explain the high reproducibility of the score in different settings and irrespective of the experience of the examiner. The AIR score has been recommended in two recent reviews with a reference to its usability and diagnostic performance [16, 17]. It has been compared with the Alvarado score in 11 studies and performed better in 10 of them [1, 18–26]. It has been prospectively validated in patients with suspicion of appendicitis in 12 previous studies [1, 10, 18–21, 23, 26–30], in most cases with similar results to the present study.

The AIR score was designed with a focus on ruling out patients with complicated appendicitis from the low risk group. These patients can safely be observed as outpatients with planned repeat examination. The few cases with complicated appendicitis in this group (0.7%) were diagnosed at the repeat examination after observation. A large proportion of the patients can thus be saved the costs of further diagnostic workup or hospital admission. This may also allow some patients with mild appendicitis to resolve spontaneously with no treatment [31].

Another aim was to identify patients with high probability of appendicitis that need an urgent surgical evaluation and a probable abdominal exploration. Some 9.2% of the patients are classified as high probability with a prevalence of appendicitis of 84%, of which the majority had complicated appendicitis (66%). One-third of all patients with complicated appendicitis was assigned to this group. The PPV was very high in patients aged <15 years (96%) and in men (89%). This may motivate an abdominal exploration with no further diagnostic work-up as imaging cannot rule out appendicitis in patients with high probability of appendicitis and a differential diagnosis is less likely. In women and patients aged ≥40 years, a diagnostic imaging may however be indicated due to the lower PPV (84% and 83%, respectively).

Imaging can identify differential diagnoses which may need further treatment or work-up. This is more common in older patients. In the present study, one patient with alternative diagnosis needing surgery was diagnosed in the low probability group and two patients in the high probability group.

Monitored in-hospital observation with repeat examination is the traditional management that has stand the test of time. In the intervention part of the STRAPPSCORE study, the patients with an intermediate AIR-score were randomised to early imaging or a period of observation followed by repeat scoring and selective imaging. We found no advantage of routine diagnostic imaging compared with observation and selective imaging [10].

The strength of the present study is its size and prospective, multicentre design, which verifies the validity of the AIR score in various settings and in the hands of physicians with varying experience. The score performed better in patients below age 15 years and in women, which are regarded as especially challenging. This support that the AIR score is applicable to all patients with suspected appendicitis irrespective of age or sex.

A weakness is the missing values of which the proportion of neutrophils was the most frequent. This reflects the logistic difficulties when introducing new methods in emergency departments with involvement of many actors that are constantly changing over time. The reported estimates should however be valid with low probability of bias as we used multiple imputation [32].

The data were collected between 2009 and 2012. Some may question if these data are still valid. However, the criteria for the appendicitis diagnosis (transmural neutrophil invasion or imaging suggesting appendiceal abscess or phlegmon) has not changed since 2012. The seven variables used in the AIR-score are still all used routinely to the same extent as in 2012. There is no new laboratory examination reported that have replaced the variables included in the score. The association of the AIR-score with the appendicitis diagnosis have therefore not been influenced by the time that has passed. The sensitivity, specificity, and discriminating capacity for at least complicated appendicitis should be valid also today in all the subgroup analyses.

However, the quality of diagnostic imaging has improved since 2012 and its usage has increased. As a consequence more cases of mild appendicitis that previously was allowed to resolve undiagnosed are now detected, as shown by an increasing incidence rate of uncomplicated appendicitis in recent decades. In the previous report of the randomised trial comparing immediate imaging with an observation period followed by selective imaging, we thus found more patients diagnosed with mild appendicitis in the imaging arm which may have resolved undiagnosed in the observation arm [10]. As we have followed up all cases, we can with confidence claim that we have not missed any patient needing treatment.

The effect of this could be a lower sensitivity for appendicitis at the low cut off. However, throughout the manuscript, we emphasise that the aim is to identify patients with complicated appendicitis with high sensitivity, whereas we do not aim at ruling out patients with mild appendicitis at the low cut-off point. We therefore suggest planned re-examination of the patients with low probability. We think this is certainly valid also in this era.

This large external validation of the AIR score verify the validity and replicability of the AIR score but shows a need to adjust the originally proposed cut-off point for the low probability. It performed especially well in children and women which are regarded as the most challenging groups for diagnosing appendicitis. The score can be used as a decision support for a risk-stratified management adapted to the probability of appendicitis. This may help minimising unproductive hospital admissions and abdominal explorations and in selection of patients for urgent surgical evaluation and diagnostic imaging.
